# Case study on force compliant robot arm controller for nasopharyngeal swab insertion

**DOI:** 10.1038/s41598-025-06032-7

**Published:** 2025-07-02

**Authors:** Peter Q. Lee, John S. Zelek, Katja Mombaur

**Affiliations:** 1 Systems Design Engineering, University of Waterloo, 200 University Avenue West, Waterloo, Canada; 2 Mechanical and Mechatronics Engineering, University of Waterloo, 200 University Avenue West, Waterloo, Canada; 3 Optimization and Biomechanics for Human-Centred Robotics (BioRobotics Lab), Institute for Anthropomatics and Robotics, Karlsruhe Institute of Technology, Karlsruhe, Germany

**Keywords:** Biomedical engineering, Mechanical engineering

## Abstract

The nasopharyngeal (NP) swab sample test, commonly used to detect COVID-19 and other respiratory illnesses, involves moving a swab through the nasal cavity to collect samples from the nasopharynx. While typically this is done by human healthcare workers, there is a significant societal interest to enable robots to do this test to reduce exposure to patients and to free up human resources. The task is challenging from the robotics perspective because of the dexterity and safety requirements. While other works have implemented specific hardware solutions, our research differentiates itself by using a ubiquitous rigid robotic arm. This work presents a case study where we investigate the strengths and challenges using compliant control system to accomplish NP swab tests with such a robotic configuration. To accomplish this, we designed a force sensing end-effector that integrates with the proposed torque controlled compliant control loop. We then conducted experiments where the robot inserted NP swabs into a 3D printed nasal cavity phantom. Ultimately, we found that the compliant control system outperformed a basic position controller and shows promise for human use. However, further efforts are needed to ensure the initial alignment with the nostril and to address head motion.

## Introduction

From the outset of the COVID-19 pandemic, the appeal to applying robots in the place of human healthcare workers has increased substantially. A major occupational hazard for healthcare workers is contracting illnesses from the patients they are treating; especially via highly contagious airborne spread illnesses like COVID-19. Robots deployed in healthcare have the advantage of being immune to illnesses, and would thereby be useful to protecting the healthcare workers and preventing downtime^1^. In cases where training consistency is a concern^2^, robotics can provide a way to standardize care. However, close-contact healthcare tasks are challenging workspaces for robots because of safety considerations and the need to meet medical objectives with the constraints of the robotic hardware.

Consequently, the research in this manuscript targets the task of nasopharyngeal (NP) swab sample collection using robots. The task involves inserting a thin, flexible swab through the nasal cavity until it reaches the nasopharynx at the anterior (just above the back of the throat). In terms of diagnosing COVID-19, many clinical studies indicate that NP swabs are considered more sensitive than other collection methods^3,4^. The task is challenging because the swab has to navigate around anatomical obstacles in the nasal cavity; namely the nasal septum (the wall that divides the left and right passages of the nose) and the inferior turbinates (the curved bony structures). The ideal path for the swab to travel is between the nasal palate and the inferior turbinates^5^ (example also later shown in Fig. 4); deviation from this path is unlikely to reach nasopharynx and can cause discomfort or injury if the swab impacts sensitive anatomy such as cribriform plate and the various nerve clusters in the nasal cavity^6^. From a control perspective, it is an interesting task because once the swab enters the nose, it becomes visually unobservable, so any adjustments must be based on measured forces applied to the swab.

**Fig. 1:**
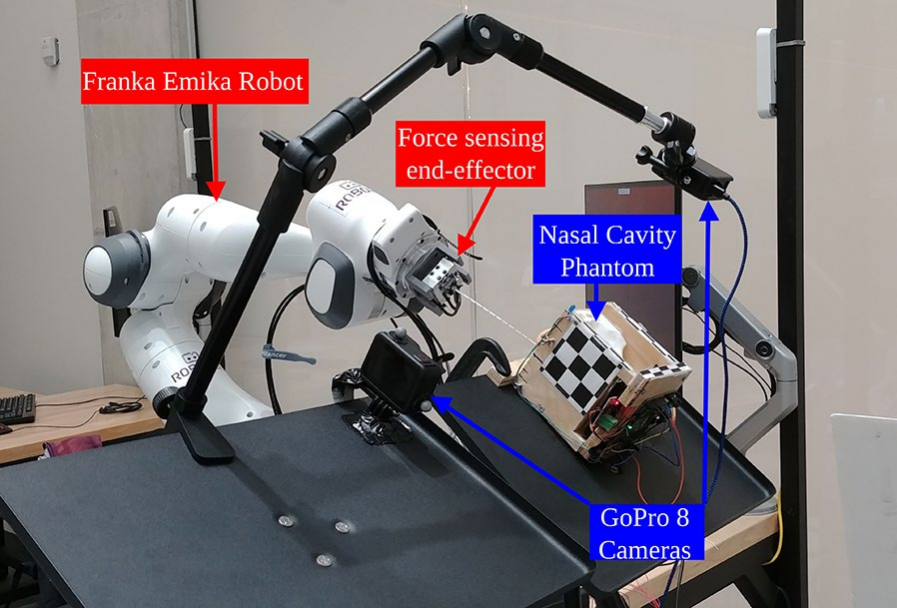
Nasal cavity apparatus arranged next to the Franka Emika Robot arm with the proposed force sensing swab end-effector attached. Two GoPro cameras are used to observe the outcome of the insertions and are not part of the control loop.

Applying robots to different types of swab sampling tasks has received increasing attention from the robotics and biomedical engineering community. Park et al.^7^ conducted a study where the forces of a practitioner were recorded using a handheld instrument on a phantom. Hwang et al.^8^ implemented a visual-servo and control system, which uses deep-learning models to detect the nose and guide the robot to more accurate positioning at the nostril prior to insertion. Li et al.^9^ also implemented visual-servo methods for NP swabs using a hierarchical decision network strategy. Li et al.^10^ designed a 3-DOF robot with an endoscopic camera to perform oropharyngeal swab tests via teleoperation. Several fully automated platforms for oropharyngeal culture collection and storage have been proposed^11,12^. These platforms utilize vision-based control algorithms, enabled by the visibility of the oropharynx through the open mouth, in conjunction with force-sensitive control loops. Chen et al.^13^ designed another teleoperated soft-rigid hybrid robot that features a compliant fiber Bragg grating integrated manipulator to adapt to disturbances during nasopharyngeal or oropharyngeal swab tests. Maeng et al.^14^ created a custom NP sampling robot featuring a remote center of motion mechanism in order to compensate for sudden forces by the patient during the procedure. Zhang et al.^15^ created a platform centred around a humanoid dual-arm robot for NP swab sampling. Their system utilized a combination of RGB-D cameras, LIDAR scanners, and a force-torque sensor to perform the task on humans, with results indicating it was very effective at gathering PCR samples. Haddadin et al.^16^ created a system to collect shallow nose and oropharyngeal cultures with a Franka Emika robot for screening COVID-19, but does not appear move far enough through the nasal cavity to reach the nasopharynx (c.f. Lim et al.^17^). There are also some commercial attempts at robotic swabbing. Lifeline robotics (Odense, Denmark) have “The Swab Robot” to perform oropharyngeal swabbing using a UR5 robot. Brain Navi’s (Zhubeai, Taiwan) “Nasal Swab Robot” uses a UR5 robot to perform NP swabs based on a 3D visual scan of the face.

Many of the developments listed above have integrated or developed custom robotic hardware in order to do the swabbing test. Unlike previous studies that developed custom hardware, our research explores the use of a single collaborative arm for NP swab sampling, with a hardware configuration that is potentially compatible with other close-contact healthcare tasks needing force regulation (e.g., temperature testing, needle work, sample collection). Specifically, we look to use a rigid manipulator robot to autonomously collect NP swab samples with an end-effector that could be adapted to manipulate the same instruments that human workers typically use. This aspect would be particularly beneficial from a clinical perspective, as it would allow the platform to perform the task without requiring special materials, which would lead to easier adoption and reliability. Furthermore, the availability of collaborative robotic arms in the market would facilitate easier implementation in healthcare settings. The constraints from this scenario provide an interesting challenge because the contact forces in these tasks would need to be regulated by the control law rather than relying on mechanical compliance from specifically designed manipulators. However, the specific capabilities required for a collaborative robot to adequately perform the NP swab test have not been thoroughly investigated or identified in previous research. With respect to these challenges, we contribute by conducting a case study to examine the suitability of a robotic arm in this scenario using an admittance control system. We first discuss the limitations of relying on the built in torque sensors to sense feedback from the swab, which we resolve through designing a low-cost, high accuracy force sensing end-effector that fits onto the flange of a collaborative robotic arm. We then describe our design of a system featuring a force-feedback torque control law to execute the insertion stage of the NP swab test. Finally, we engage in experiments with the robot on a 3D printed phantom of a human nasal cavity and compare its performance with a baseline position controller to determine the effectiveness of the setup for performing NP swab tests.

## Materials and methods

The hardware used in this study consist of the collaborative robotic arm platform (Section 2.1), the custom designed force-sensing end-effector where the NP swab is mounted (Section 2.2), and the nasal cavity apparatus for validating our methods (Section 2.3). An overview of the proposed control system is given in (Section 2.4), which consists of a force filtering subsystem (Section 2.5), trajectory generation (Section 2.6), control law (Section 2.7), and an observer for determining completion of swab insertion (Section 2.8).

### Collaborative robot arm

The robotic platform used in this work is the Franka Emika Robot “Panda” arm (Franka Emika, Munich Germany) and is shown in Fig. 1. We assume the robot to follow the second-order dynamics^18^

1 $$\begin{aligned} \tau = M(q) \ddot{q} + C(q,\dot{q}) \dot{q} + g(q), \end{aligned}$$

where *M* is the mass inertia matrix, *C* is the Coriolis effects matrix, *g* is the gravitational vector, $\tau$ is the torque vector, $\ddot{q},\dot{q},q$ are the joint acceleration, velocity, and position vectors. The arm has 7-DOF and is designed as a collaborative robot that is meant to be used to perform tasks in conjunction with humans. While we chose to use the Panda due to its ubiquity, it should be noted that our work could generalize to similar collaborative arms on the market.

### Force sensing end-effector

**Fig. 2:**
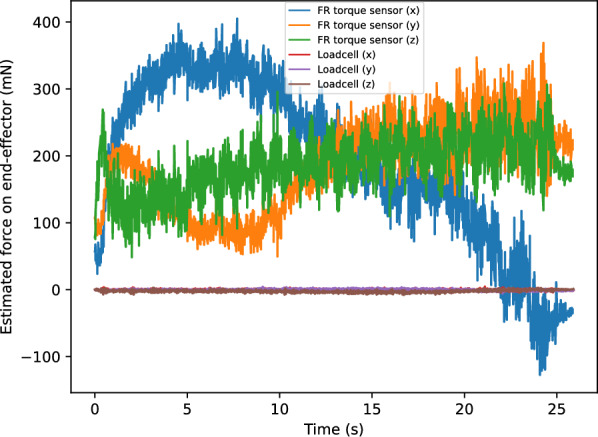
Comparison of external force estimation methods evaluated with the arm moving through free space without any load. The underlying Franka Emika (FR) system estimates task space load by projecting the measurements from its seven torque sensors and has an unsuitable amount of noise and drift. The proposed loadcell has much lower noise and is much more suitable for responding to forces transmitted by the swab.

**Fig. 3:**
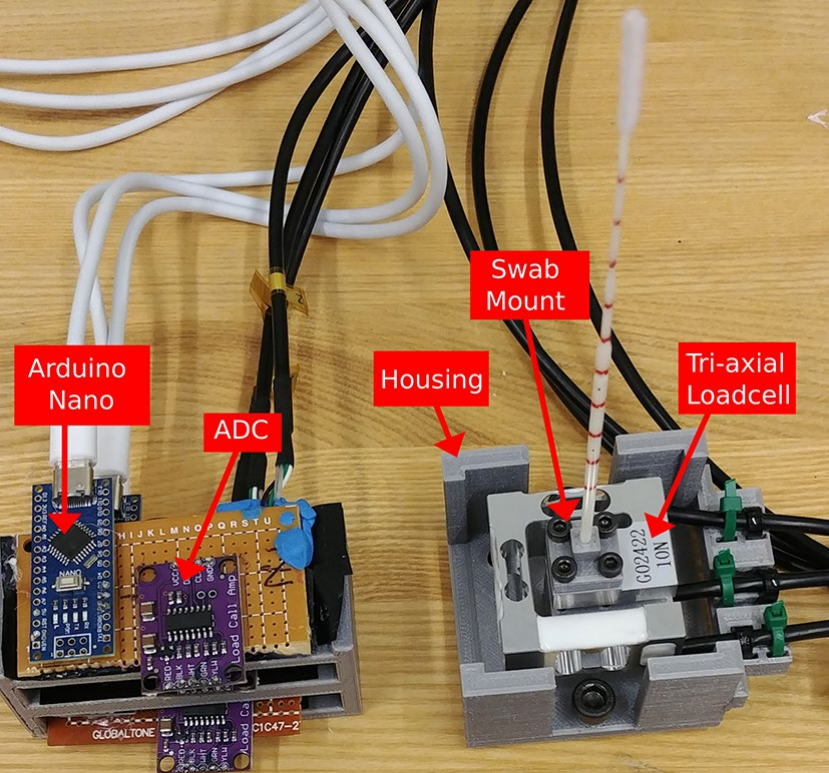
Components of the custom end-effector. Left: Wheatstone bridge amplifier and digital conversion circuit. Right: the tri-axial loadcell interfaced with its housing and swab mount. Note that the red stripes on the swab are labelled to gauge distance in experiments, but do not have any functional purpose related to the controller.

While the Franka Emika control system has a method to estimate the external forces applied to the end-effector by projecting the measurements of the seven torque sensors on the joints, the measurements are too insensitive for reacting to the small forces applied to the NP swab. Fig. 2 demonstrates this by showing the estimated forces as the arm moves, without an end-effector, through empty space. There is significant noise in the estimates for the Franka Emika system, reaching 40 mN to 60 mN standard deviations among the three axes. Since persistent model errors result in non-stationary drift as the arm changes configuration, the uncompensated weight of the links gets erroneously interpreted as external torque. Under these conditions, it is clear that any low magnitude forces that would be transmitted through a swab would be swallowed up by these disturbances if we were to solely rely on the built-in torque sensors. Therefore, we designed a custom end-effector to mount a NP swab and sense the 3 axial forces applied to it. We integrate a GPB160 10 N capacity tri-axial strain gauge loadcell (Galoce, Shaanxi, China) onto a 3D printed housing to be mounted onto the Panda’s flange. A 3D printed mount that fits the end of an NP swab is affixed to the exterior facing side of the loadcell (The 3D print STL files are available in the supplementary data). We adopt a fairly simple electronics setup: each of the leads from the load cell axes are soldered into an HX711 Wheatstone bridge amplifier set to 80 Hz mode that is interfaced with an Arduino Nano. An image of these components is shown in Fig. 3. A ROS node was created to publish the values sensed from the three axes via the controlling computer. Overall, the entire cost of the materials for the sensorized end-effector was less than $300 USD, which makes it an affordable solution, provided a robot arm is already available. The base of the end-effector was 3D printed with PLA to screw into the Panda’s flange (DIN ISO 9409-1-A50). This base piece could easily be adapted and reprinted to other mounting flanges on other robots. While this prototype is made with swabs as the application, we foresee that other tools could be attached to the loadcell that could enable other medical tasks.

In practice, the implementation is sufficiently sensitive because of the fine capacity of the loadcell. The electronic noise present in the signal remains quite low as well, having a standard deviation of about 1 mN that is further low pass filtered for reasons described in subsection 2.5. However, one detail that requires special attention is the impact of gravity on the readings. As the end-effector changes orientation, the forces due to gravity will need to be subtracted within the end-effector frame in order to isolate the raw forces applied to the swab. In an ideal case, we would subtract the weight attached to the loadcell. However, the wires leading from the loadcell create non-trivial effects on the readings as their tension changes with gravity.

The net force read on the loadcell can be described as

2 $$\begin{aligned} F_{net} = F + G(o) + Z \end{aligned}$$

where *F* is the external force vector applied to the swab, *G* is the force gravity vector as a the normalized direction vector $o=(o_x,o_y,o_z)$, and *Z* is the bias offset. The vector *o* represents the direction of the global vertical axis in the frame of end-effector, which is found by using forward kinematics. Thus, if not for the aforementioned sources of error, we would expect $G(o) = m*9.81 \text { m/s}*o$, where *m* is the mass of the payload. Instead, we empirically model *G*(*o*) based on a calibration routine where the end-effector is moved to nine different orientations with no external force ($F=0$). Specifically, the end-effector was rotated along independent axes so that *o* was placed between $[0,0,-1] \rightarrow [1,0,0] \rightarrow [0,-1,0] \rightarrow [0,0,-1]$. These orientations spanned all necessary orientations of *o* under the SO(3) rotational group, under the assumption that *G*(*o*) would reflect across the axes. The gravitational force is estimated with a multiple linear regression model. The regression analysis found significant effects by the non-zero terms in the $A\in \mathbb {R}^{3 \times 4}$ matrix written below, with one significant interaction term $o_y o_z$ for $G_y$.

3 $$\begin{aligned} G(o) = \left[ {\begin{smallmatrix} A_{x,x} & A_{x,y} & A_{x,z} & 0 \\ A_{y,x} & A_{y,x} & A_{y,z} & A_{y,yz} \\ 0 & 0 & A_{z,z} & 0 \end{smallmatrix}}\right] \left[ {\begin{smallmatrix} o_x\\ o_y\\ o_z \\ o_{y}o_z \end{smallmatrix}}\right] = A \left[ {\begin{smallmatrix} o_x\\ o_y\\ o_z \\ o_{y}o_z \end{smallmatrix}}\right] . \end{aligned}$$

Finally, we can solve for the parameters of the gravitational + offset model via a least squares problem over the $F_{net}$ and *o* values from the nine orientations

4 $$\begin{aligned} \min _{A,Z} {|| F_{net} - (A\begin{bmatrix} o_x&o_y&o_z&o_{y}o_z \end{bmatrix}^{\top } + Z) ||}^2 \end{aligned}$$

The fit for this calibration procedure is quite good, achieving $R^2 > 0.9999$, which is equivalent to about 1 mN error across the different orientations. The low error rate indicates the compensation method can effectively eliminate the gravitational effects.

### Nasal cavity apparatus and experiment setup

**Fig. 4:**
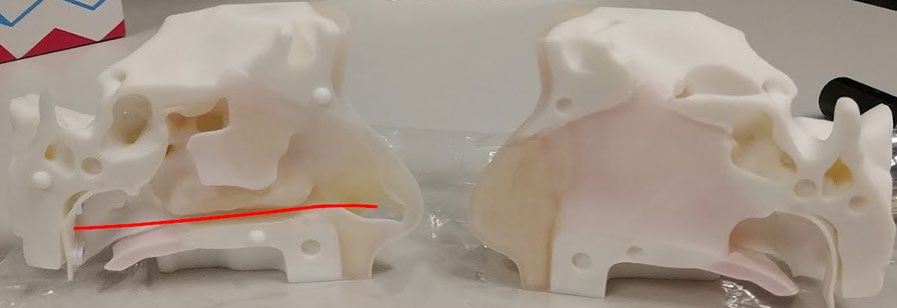
Nasal cavity phantom used for validation experiments. The red line highlights the path to reach the nasopharynx from the nostril.

The other component used in this work is the nasal cavity phantom that is used to evaluate the proposed methods. Ideally, we would want to validate our methods with biological tissue. However, the nasal cavities of most animals have distinct morphologies compared to humans, and the tissue of cadavers would lose most of the physical properties of living tissue. As substitute, we used the 3D printed nasal cavity phantom (shown in Fig. 4) designed by Sananès et al.^19^ that was printed with a PolyJet 3D printer. The apparatus is printed with the rubber-like material “Agilus 30” to provide a more genuine analogue to the fleshy mucosal tissue in the nasal cavity than more typical rigid 3D printing materials. The figure also shows the ideal path a swab should follow, leading from the nostril to the nasopharynx. The phantom is placed into an open fitting container and was augmented by gluing a force sensing resistor (FSR) onto the nasopharynx. The apparatus was clamped to a tripod so that it could be re-positioned and reoriented freely. Two GoPro Hero 8 cameras were used to record the experiments and were aligned with an adjacent tripod such that they faced the apparatus in the top-down and sideways directions. Fig. 1 shows a photo of this setup.

### Control system

**Fig. 5:**
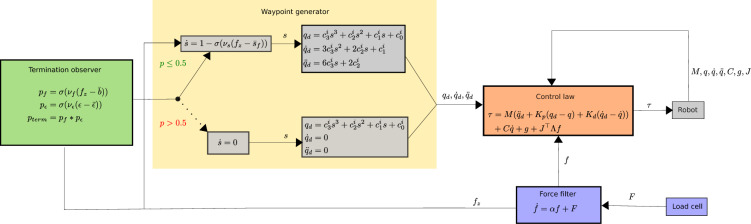
Block diagram for the proposed NP swab insertion system.

The proposed system consists of four components: a force filter for the loadcell readings, a waypoint trajectory generator, the torque control law, and an observer to determine when the insertion procedure is completed. A block diagram summarizing the interaction between these components is shown in Fig. 5. Each of these components are described in the following sections.

The software to implement this system was written in C++, running on a laptop with an Intel i7-10750H 2.60 GHz CPU and the Ubuntu 20.04 operating system with the RT_PREEMPT kernel patch. The control loop runs at a frequency of 1000 Hz, communicating with the Franka Control Interface (FCI) via ROS over Ethernet to transmit commands and receive the robot state from the control box, while measurements from the loadcell are taken at 80 Hz. While the difference between the controller frequency and force measurement may appear to conflict, this is not a fundamental issue. There is inherent latency between the control system (on the PC) and the robot itself, as the control box performs filtering and rate-limiting operations on the transmitted commands. Thus, this latency would limit reaction to external forces, even if measurements were performed at a higher rate. Ultimately, the impact of the system latency is mitigated through the filtering subsystem that is described in the following section.

### Force filtering

As described in subsection 2.2 the loadcell force readings already have a low level of noise. However, it is still desirable to filter the force signals because of the aforementioned system delays in the FCI. Using direct force measurements with the system delay causes the swab to constantly rebound between the walls of the nasal cavity, as the robot reacts latently to the previously issued commands. This results in jerky and unstable motion as the swab rebounds from intermittent contacts of the walls of the nasal cavity, which is undesirable and dangerous behaviour to have within the nasal cavity. We therefore apply the following filter to smooth the system’s reaction to force:

5 $$\begin{aligned} \dot{f} = -\alpha f + \alpha F, \end{aligned}$$

where *F* is the raw force measurement coming from the loadcell, *f* is the filtered signal that will be used in the control system, and $\alpha \ge 0$ is the response rate. This is a continuous implementation of an exponential moving average filter. Ultimately, we want the filter to attenuate sudden changes in force and respond to consistent levels of force. Having a delayed response in *f* is ideal because this encourages the controller to make gradual adjustments and discourage jerky and unstable motion. In our experiments we chose $\alpha =1$ because it provided the response we desired.

### Trajectory waypoint generation

From our previous work^20^, we studied the deformation of NP swabs as they were inserted through the nasal cavity and found the optimal linear trajectory to insert at to minimize swab deformation. We build on these ideas by projecting a linear task-space trajectory to follow in the arm’s workspace. We take insertion at a 28 degree decline angle (c.f.^21^) over 20 cm, selecting 32 points along this line and solving for continuous joint configurations (*q*) with inverse-kinematics using RBDL-Casadi^22^. Cubic splines were fit between each of the points, having coefficients $\{c^i\}$ with respect to the path parameter *s*. The desired nominal joint waypoints are computed as

6 $$\begin{aligned} \begin{aligned} q_d&= c^i_3 s^3 + c^i_2 s^2 + c^i_1 s + c^i_0\\ \dot{q}_d&= 3 c^i_3 s^2 + 2 c^i_2 s + c^i_1\\ \ddot{q}_d&= 6c^i_3 s + 2c^i_2, \\ \end{aligned} \end{aligned}$$

where $c^i$ is the coefficient vector that corresponds to the spline within the domain of *s*. These splines are all computed offline, and the coefficients are used during runtime to determine the associated goal positions, velocities, and accelerations.

We design the progression of *s* with respect to time as

7 $$\begin{aligned} \dot{s} = 1 - \sigma ( \nu _s(f_z - \bar{s}_f)), \end{aligned}$$

where $\sigma$ is the sigmoid function; the scalars $\nu _s$ and $\bar{s}_z$ influence the scale and offset of the sigmoid response. The purpose of this formulation is to allow the nominal trajectory to respond to disturbances via the force reading $f_z$, which faces the direction the swab will be moving along. When there is little $f_z$, the sigmoid remains unsaturated and the trajectory proceeds normally. When there is high $f_z$, the sigmoid becomes saturated and the trajectory slows down to allow time for the controller, described in the subsection 2.7, to make adjustments and respond to the disturbance. We set $\bar{s}_f=0.33$ and $\nu _s = 12$; based on experimentation, these values allowed the trajectory to slow down on when it encountered early contacts without stopping completely.

### Torque control law

The task of the control law is to strike a balance between two things: a) following the nominal trajectory waypoints, $q_d,\dot{q}_d,\ddot{q}_d$ and b) adjusting to contact forces *f* that are applied to the swab. As a result, we designed the admittance controller based on a computed torque control law^23^ using feedforward acceleration with position, velocity, and force feedback

8 $$\begin{aligned} \tau = M(q) ( \ddot{q}_d + K_p (q_d - q) + K_d (\dot{q}_d - \dot{q})) + C(q,\dot{q})\dot{q} + g(q) + J^{\top } \Lambda f. \end{aligned}$$

Here, $\ddot{q}_d,\dot{q}_d,q_d$ and $\dot{q},q$ are the acceleration, velocity, and positions for the nominal joint waypoints and the actual joints, respectively. *M* is the mass inertia matrix, *C* is the Coriolis matrix, *g* is the gravitational vector, *J* is the $7\times 3$ joint-euclidean space Jacobian matrix, $K_p=\text {diag}(600,600,600,600,600,600,50),K_d=\text {diag}(30,30,30,30,30,30,5)$ are diagonal gain matrices for position and velocity. One can see how the first line is used to drive the trajectory towards the nominal waypoints. The acceleration term is fed forward in the control loop, and the position and velocity terms are used as feedback to correct for errors, which is generally understood to provide better tracking than PD control on its own^23^. The gravitational and Coriolis forces are included to counteract these effects from Eq. 1. Finally we amplify the small scale *f* with $\Lambda =\text {diag}(450,450,45)$ to promote motion and map it to joint torques with $J^{\top }\Lambda f$. The purpose of this component is to shift the trajectory away from contact forces, with the goal of correcting for misalignment. The gain values for $K_p$, $K_d$, and $\Lambda$ were chosen with trial and error and could be refined in the future.

### Termination observer

The last component of the system is an observer to estimate when the swab has reached the nasopharynx, during which the insertion stage should be terminated. Typically this would be followed with rotating the swab to collect samples, but this motion is deferred for this paper. With the absence of additional sensors, the controller makes this determination based on two sources of information a) the Z-axis force and b) the total positional displacement of the end-effector. Fuzzy logic^24^ presents a robust way of making this decision, which has seen use in situations where decisions rely on measurements that are linked to a state with uncertainty^25^. The fuzzy model we use is

9 $$\begin{aligned} p_f = \sigma (\nu _f(f_z - \bar{f}_z))),\ p_\epsilon = \sigma (\nu _\epsilon (\epsilon - \bar{\epsilon })),\ p_{\text {term}} = p_f p_\epsilon , \end{aligned}$$

where $\sigma$ is the sigmoid function and the termination decision $p_{\text {term}}$ is activated when both the terms for force, $p_f$, and position, $p_\epsilon$, are sufficiently saturated. The force sigmoid $p_f$ saturates when $f_z$ rises, which we expect to happen when the swab tip makes contact with the nasopharynx. Likewise, the position sigmoid $p_\epsilon$ saturates when the total displacement of the end-effector position from its starting position, $\epsilon$, is high enough so that contact with the nasopharynx is possible. The parameters $\nu _f$, $\nu _\epsilon$, $\bar{f}_z$, and $\bar{\epsilon }$ control the scale and intercept of the sigmoid activations. We set the threshold for $p_{\text {term}}$ at 0.5, where if at any point $p_{\text {term}}>0.5$, the trajectory halts. We set $\bar{f}_z =0.167$, $\nu _f = 30$, $\bar{\epsilon } = 0.085$ m, and $\nu _\epsilon = 40$. Intercept $\bar{\epsilon }$ was chosen based on the nasal cavity geometry as we expect the swab to travel at least 9 cm. The other parameters were chosen by trial and error; these values could also be refined because we typically saw $p_f$ become saturated before $p_\epsilon$.

**Table 1:** Contingency table comparing the rates of success between the two controllers. A chi-square test results in a p-value = $1.68 \times 10^{-4}$, indicating that the proposed method has a significantly higher success rate.

Controller	Success	Failure
Proposed	33	9
Baseline	16	26

**Table 2:** Summary of measured forces applied during the swab over all trials. Peak force and the forces averaged over the duration of the insertion are compared between the two controllers using paired t-tests.

	Proposed(mN)	Baseline(mN)	p-value
Avg. Force	$250 \pm 112$	$375 \pm 210$	$4.6 \times 10^{-5}$
Peak Force	$1064 \pm 325$	$1347 \pm 516$	$5.3 \times 10^{-4}$

## Experiments

The swabs that are used for NP culture collection typically have compliant properties that allow the swab to deform within the nasal cavity. These properties are beneficial because, when properly aligned, it allows the swab to navigate through the nasal cavity despite its complex geometry. However, it cannot be expected for the nominal alignment to always be maintained, as placing the swab at the nostrils will require visually guided systems which will have placement variance and any patient would have natural head motion as well. Consequently, the goals of the experiments in this study are to examine if a controller with force-reactive properties will lead to greater success and better performance compared to relying on the compliant properties of the swab. Our experiments will be comparing the performance of the proposed controller to a baseline controller, which relies solely upon the compliant properties of the swab, in terms their ability to insert the swab and reach the nasopharynx of the nasal cavity apparatus that is perturbed at different orientations. The baseline controller uses the same control law as the proposed controller (Eq. 8) but sets $\Lambda =0$, ignores force for determining waypoints (i.e., $\dot{s}=1$), but still uses the same termination condition Eq. 9.

**Fig. 6:**
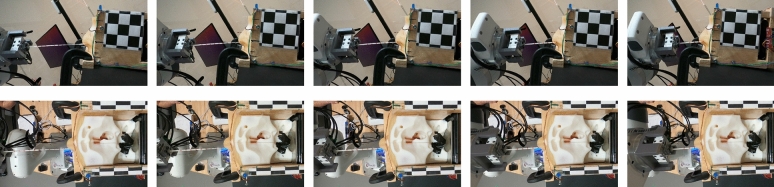
Side and top frames of a successful insertion taken by the two GoPro cameras.

**Fig. 7:**
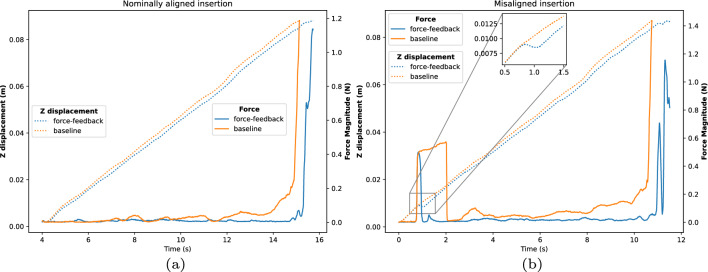
Comparison of total observed forces and the accompanying displacement along the insertion axis for both versions of controllers recorded from the start of motion until the termination condition is reached, which is marked by the impulse of force as the swab reaches the nasopharynx. (**a**) forces encountered during a nominal insertion angle. (**b**) forces encountered during a misaligned insertion. Notice how the proposed controller is able to adjust and minimize a collision that occurs early in the insertion.

The insertion trials were done by moving the arm to the first waypoint, after which the apparatus was manually positioned in front of the swab. The controllers were evaluated in a paired manner, where the force-feedback controller would execute, then after resetting the arm to the starting position without moving the apparatus, the baseline controller would execute. We executed a total of 41 of these paired trials. Between each of these trials we would move the apparatus to a unique position and angle in order to add variance and also to simulate pose estimation errors that would occur if a visual system were guiding the initial placement. The apparatus was perturbed in such a manner that the tip of the swab would be placed at the nostril, but was oriented within a range of about $\pm 5$ degrees of pitch and ± 10 degrees of yaw. Fig. 6 shows frames from top and side videos that were taken during a successful insertion. Typically, the insertion would take about 15 seconds, but the total time varied depending on the disturbances encountered from the misalignment. The video attached in the supplementary data includes a couple of recordings taken during the insertion trials.

**Fig. 8:**
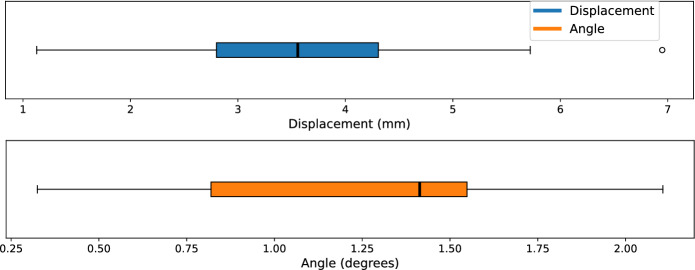
Boxplots showing the difference in the final end-effector position and angle between the proposed and baseline controllers on trials where both controllers successfully reached the nasopharynx. The black line indicates the median, while the box and whiskers occupy 1 and 1.5 times the interquartile range.

Data recorded in the trials consists of videos from the two cameras and the published robot states, loadcell, and FSR values in the ROS server. Success was defined based on if the swab reached the nasopharynx and was largely determined by visual examination of the swab because the threshold of force needed to activate the FSR was too high. As shown in Table 1, the trials resulted in a higher insertion success rate of 78.6% for the force-feedback controller compared to the baseline controller with 38.1%. Comparing these outcomes with a chi-square test shows that the result is statistically significant $p=1.68 \times 10^{-4} < 0.05$. It is also interesting to examine the amounts of force applied to the swab, as this can be used as a proxy to the quality of the insertion and to the patient’s comfort. The forces that were sustained on the loadcell during trajectories are shown in Table 2. In a paired manner, the force-feedback controller sustained lower force than the baseline controller, but there was wide inter-trial variance based on the swab’s initial placement. Fig. 7 shows two graphs comparing the forces and displacement observed by the controllers on two different initial alignments, which run until the termination observer triggers from sufficient force and displacement. The left graph shows the forces for a nominally positioned swab, and the right graph shows the forces for a misaligned swab. In the latter case, the force-feedback controller is able to adjust itself and sustain less persistent force compared to the baseline. From examining the final end-effector pose from forward kinematics of the recorded joint angles, the impact of the force feedback is also apparent: the trajectory from the left graph had a displacement of 2.4 mm and 0.77 degrees, while the right graph was altered by a larger factor with a displacement of 6.9 mm and 2.1 degrees. The proposed controller clearly alters the trajectory based on the force feedback stemming from misalignment. Fig. 8 shows the distribution of the differences in final pose for trials where both controllers were successful, with a median end-effector pose differing by 3.6 mm and 1.4 degrees.

## Discussion

As we showed in Table 1, the force-feedback controller was significantly more successful than the baseline controller according to a Chi-squared test, suggesting that incorporating force in the control loop enabled the insertion trajectories to be successfully altered to non-ideal alignments with respect to the phantom. The lower sustained forces in the force-feedback controller also indicates that it performed better than the baseline. The peak force was about 0.4 N higher than the expert practitioner’s from Park et al.^7^, which could stem from physical differences in the phantom or from them only using nominal insertion angles, but could also indicate that our controller parameters in Eq. 7 and Eq. 9 could be better tuned in the future. Hence, the outcome of these trials show that there is certainly benefit to incorporating such a force-sensed based compliant control system for the proposed robotic arm setup that will increase insertion success and patient comfort versus a closed loop position controller.

Generally the compliance of the swab meant that there was significant allowance for the swab to be misaligned and still make it to the nasopharynx. Qualitatively, we notice that the pose and ease of insertion seemed to correlate with the findings from previous work^20^. Insertion angles that were oriented towards the septum were generally more successful than those oriented away from the septum. Being positioned away from the nasal vestibule wall also seemed to be important for avoiding the wedging state and to reduce strain on the swab. While characteristics may differ for individual anatomy, we plan to take these observations into account when we design the vision guided positioning system for the initial placement of the swab in future work.

**Fig. 9:**
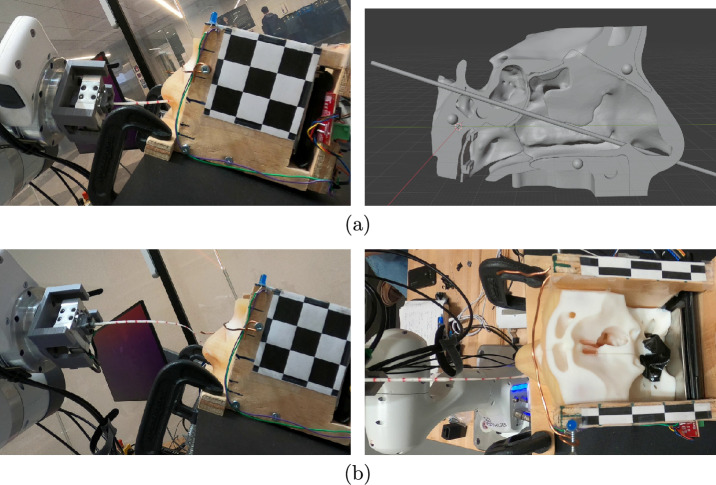
Two types of failure states observed in the trials: (**a**) The elevation angle for the insertion is too high, resulting in it travelling through the wrong passage in the nasal cavity. The proposed compliant controller prevented these states. (**b**) The swab becomes wedged before entering the nasal cavity. This state occurred with both controllers.

It is insightful to examine the cases where the insertion failed for either controller. There were two types of failure states that occurred during insertion. The first type of failure was unique to the baseline controller, which failed to reach the nasopharynx because the elevation angle of the swab was too high (see Fig. 9a). Typically, this transpired when the swab was positioned too low and then became levered into an excessive angle where it deflected off the sphenoid sinus, which would likely be more uncomfortable and have higher chance of complications for a human patient. The force-feedback controller was able to adjust the trajectory to avoid this levering effect because of the adjustments the force-feedback produced. The swab becoming stuck and being unable to enter the nasal cavity, as shown in Fig. 9b), was the second type of failure. This failure state was the result of the swab becoming wedged on the nasal vestibule because of poor alignment and appeared with both the force-feedback and baseline controller. This highlights that implementing a strategy to detect contact within the nasal vestibule and to compensate the trajectory of the swab during the first centimetre of insertion as the main recommendation to improve insertion success.

In terms of future work, one of the major aspects that would need to be resolved is enforcing guarantees for safety. One such area is providing guarantees for the stability of the controller. While the force filter in subsection 2.5 helped stabilize the response compared to using unfiltered values, there were still some cases where oscillations were present as the swab reached the nasopharynx. An untested scenario is the controller’s response to the natural motion that a human patient would have during the procedure. From the hardware design, it is important to eventually build in a mechanism to detach the swab from the robot in cases where extremely high forces are detected or when the participant wants to abort the procedure. Finally, although the force-feedback controller was able to handle non-ideal insertion poses, it may be fruitful to investigate if applying an estimator that directly estimates pose errors via the force feedback could result in improvement, particularly for resolving the wedging failure case.

While the experiments demonstrated the necessity of using force-feedback for a swab insertion controller, they had several limitations that could be addressed in future studies. One aspect to improve is better quantifying and controlling the pose offsets of the nasal cavity from the swab. The perturbations we added during the experiments were implemented manually in an ad-hoc manner. With appropriate motion capture systems, it would be useful to collect statistics on the range of offsets that can be tolerated by the insertion controller. This would help define the required accuracy of visual systems that would place the swab at the nostril. Additionally, incorporating more nasal cavity models in future experiments would ensure that the system is robust to different nasal cavity morphologies among the population. Rather than having the nasal cavity on a stationary platform, placing it on an actuated platform would allow for head motion simulation that would further validate the robustness of the controller.

The control law implementation is another area that can be improved in the future. As was previously mentioned in Section 2.4, the FCI requires communicating via Ethernet with a control box to issue commands and measure the robot’s state. Although the rate of packet transmission is nominally 1 kHz, the control box adds additional latency between the PC and hardware due to rate limiting and filtering. From a perspective of torque control, the latency issues are compounded by the fact that the control law (Eq. 8) operates with outdated robot state parameters, resulting in further inaccuracies. Model errors and friction also limit tracking performance, which could not be fully compensated for by increasing gain parameters, due to the tendency to invoke instability. Consequently, we believe that the system could be improved by switching the control interface from torque control mode to velocity control mode because a first order system will be more robust to latency than a second-order system. This would also allow model errors to be handled to the control box, which is better suited to compensate due to its direct interface with the motors and joint encoders.

## Conclusion

In this work, we proposed a scenario where a standardized fixed rigid arm robot performs NP swab sampling using a compliant control system. To investigate this, we designed a minimal force sensing end-effector and integrated it into a Panda arm that could be adapted to similar rigid arms. We designed an admittance force-feedback torque control system to perform the insertion test. We performed experiments to evaluate our system on a 3D printed nasal cavity from variety of different alignment conditions and showed that the admittance controller was succeeded at a rate of 78.6% compared to 38.1% of the baseline position controller. This demonstrates that there is feasibility for a rigid arm to perform the NP swab test on people using sensitive force feedback as a modality. The compliant controller was able to compensate for some misalignment and thereby avoid some failure states, however it is clear that a full solution will require additional sensors or other strategies to adjust for more extreme misalignment and more study will be needed to evaluate the impact of head motion on the controller.

Future work will extend this research towards implementing a fully automated robotic NP sampling solution. An eye-in-hand visual servo system will be a necessary for reaching the pose in front of the nostril, prior to insertion. The other stages of the NP test (rotating the swab at the nasopharynx and extraction) will need to be implemented. As well, adding additional inputs, such as tracked visual features of the face, may be worth fusing with the force measurements as inputs to the control system to better adjust to disturbances such as motions of the head and general misalignment.

While the NP swab test is just one task a healthcare worker would do in a clinical setting, there are many other types of routine close-contact tasks that would require similar levels of dexterity and force sensitivity. Examples include other types of sample collection, medication or blood collection through needles, and skin temperature testing. Consequently, having a multi-purpose robot that could do NP swab tests could open up a multitude of different clinical tasks, and could be a boon for the healthcare system by allowing procedures to be done autonomously, enabling human resources to be reallocated.

## Supplementary Information

Supplementary Information 1.

Supplementary Information 2.

## Data Availability

Materials used in this study is included in the supplementary files.

## Code availability

The controller code and load cell interface code used in this study is included in the supplementary files.
